# Supplementary material for: DNA Methylation Analysis Identifies Novel Epigenetic Loci in Dilated Murine Heart upon Exposure to Volume Overload
Source: Int J Mol Sci. 2023 Mar 20;24(6):5885. doi: 10.3390/ijms24065885 (PMC10059258; doi:10.3390/ijms24065885)
Supplement: Supplementary file 1 [file ijms-24-05885-s001.zip › ijms-2256092-supplementary.pdf]

Supplementary Figure S1. MeDIP-qPCR primer establishment. Corresponding amplification curves are shown, which were generated by using serial dilution of DNA from 1:10 to 1,000,000.

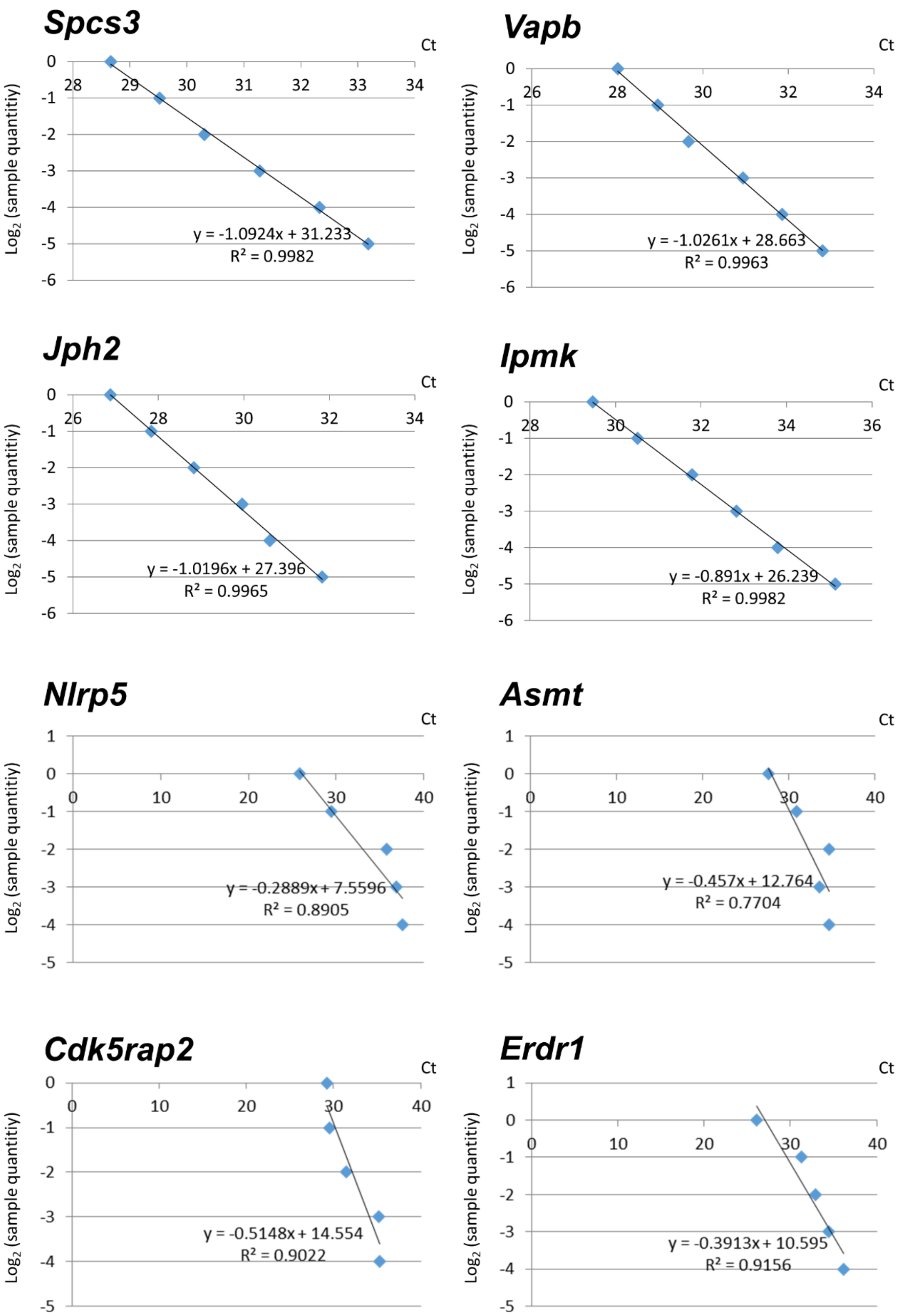

Supplementary Table S1. Echocardiographic measurements of sham and shunt mice at 16 weeks after operation

	Sham (9 mice)	Shunt (8 mice)
HR (bpm)	434 ± 7.09	471 ± 11.48
LVESD (mm)	3.559 ± 0.09	5.39 ± 0.29**
LVEDD (mm)	4.67 ± 0.06	6.36 ± 0.20***
Vol s (µl)	53.10 ± 3.27	143.69 ± 17.13**
Vol d (µl)	101.04 ± 2.87	206.92 ± 14.40**
SV (µl)	47.94 ± 3.76	63.24 ± 9.03
LVAWT (mm)	0.58 ± 0.05	0.75 ± 0.04*
LVPWT (mm)	0.602 ± 0.03	0.75 ± 0.04*
FS (%)	23.83 ± 2.00	15.41 ± 2.51*
EF %	47.40 ± 3.26	31.45 ± 4.83*
RWT	0.25 ± 0.016	0.24 ± 0.01
LV Mass (mg)	112.31 ± 8.68	232.32 ± 20.69**

HR, heart rate; LVAWT, left ventricular anterior wall thickness; LVEDD, LV end-diastolic dimension; LVESD, LV end-systolic dimension; LVM, left ventricle mass; LVPWT, left ventricular posterior wall thickness; EF, ejection fraction; FS, fractional shortening; RWT, relative wall thickness (LVAWT+LVPWT/ LVEDD). Vol d, left ventricle volume at diastole; Vol s, left ventricle volume at systole; SV, left ventricle stroke volume; Data are expressed as mean ± SEM. *$P < 0.05$; **$P < 0.01$; ***$P < 0.001$ *vs.* sham.

Supplementary Table S2. CpG methylation patterns in sham and shunt hearts at 16 weeks after OP

File	Total Reads	Aligned Reads	Unaligned Reads	Ambiguously Aligned Reads	No Genomic Sequence	Total Cs	Methylated CpGs	Unmethylated CpGs	Methylated CpHs	Unmethylated CpHs	Methylated CHHs	Unmethylated CHHs	CpG(%)	CHG(%)	CHH(%)	Mean	SD
Sham1_bismark_report	18166274	13612188	1598667	2955419	0	134941403	3750603	2167425	411611	28503974	1257642	98850148	63.60%	1.75%	1.50%	64.92%	0.031860372
Sham2_bismark_report	30663247	22344104	3248396	5070747	0	222523571	6320799	2976072	519533	47429133	1634809	163643225	68.55%	1.70%	1.50%		
Sham3_bismark_report	23840904	17600703	2279118	3961083	0	173700677	4786921	2918493	453707	36604176	1431534	127505846	62.60%	1.75%	1.50%		
Shunt1_bismark_report	21551320	16183061	1930367	3437892	0	160929710	4410031	2593680	470894	33998293	1420947	118035865	63.25%	1.65%	1.40%	66.15%	0.01758124
Shunt2_bismark_report	42890670	30424652	4603783	7862235	0	299418789	8255218	4305443	677092	62609997	2175707	221395332	66.25%	1.65%	1.40%		
Shunt3_bismark_report	11977047	6155005	4342905	1479137	0	61449315	1633857	873647	63584	12888238	240549	45749440	65.25%	0.50%	0.55%		
Shunt4_bismark_report	61311052	45414266	5534222	10362564	0	452468263	12712154	6246966	1056062	96006816	3311685	333134580	67.55%	1.70%	1.45%		
Shunt5_bismark_report	6386020	3855443	1688293	842284	0	38650919	1077208	501783	38497	8280445	143916	28609070	68.20%	0.50%	0.50%		
Shunt6_bismark_report	26241971	19517293	2262616	4462062	0	193054291	5385583	2776128	501453	40846615	1558933	141985579	66.40%	1.75%	1.50%		

Supplementary Table S3. Primer sequences used for MeDIP-qPCR and the coordinates for the amplified regions

Region	Sequences	Amplicon size (bp)
Spcs3<br>(+135 - +220)	5′-GCTTTCAGTGAATCCGCCAA-3′<br>5′-AGTGGAGGAGGCGGGTCT-3′	85
Vapb<br>(-126 - -12)	5′-CCTCCCAAGCCTCGAGAG-3′<br>5′-CAAAAGGGAGACTGCGCATG-3′	114
Jph2<br>(-133 - -50)	5′-GAAAAGGAAGCCGACCGAAG-3′<br>5′-AAGGAGAGCACACCAGACTC-3′	83
Ipmk<br>(-277 - -196)	5′-GACTCTAGACCCGCTCAGTT-3′<br>5′-CTACCGGACCAGACCTGC-3′	81

Supplementary Table S4. Primer sequences used for bisulfite-sequencing analysis and the coordinates for the amplified regions

Primer	Sequences
m*Spc3*_BS<br>(+81- +241)	5′-TTATATTTTTTGGGTTTTATTTATTGGG-3′<br>5′-AAAACCATAAAACAACTAAACAATAAAA-3′
m*Vapb*_BS<br>(-160- -28)	5′-GTTTATTGAATAGTTTTATTGGAGAT-3′<br>5′-CATACRCAAAATATTCAAAACCAC-3′
m*Jph2*_BS<br>(-151- -28)	5′-TTAATAAGGTAGTAATTTGAAAAGGA-3′<br>5′-AACTCCTTTCRTTACTTTTCTTAAAAAA-3′
m*Ipmk*_BS<br>(-351- -138)	5′-TAAGTAGGGGTATTTGAGATAAGTTTTA-3′<br>5′-ATACCCAAAAACTACCCTACTAAAT-3′

Supplementary Table S5. Primer sequences used for mRNA expression analysis by real-time qPCR

Transcript	Sequences
Dnmt1	5′-GCCATCTCTTTCCAAGTCTTT-3′<br>5′-TGTTCTGTCGTCTGCAACCT-3′
Dnmt3a	5′-GCTTTCTTCTCAGCCTCCCT-3′<br>5′-CCATGCCAAGACTCACCTTC-3′
Dnmt3b	5′-ATCCATAGTGCCTTGGGACC-3′<br>5′-CTGGCACCCTCTTCTTCATT-3′
Tet1	5′-GGGAGCTCATGGAGACTAGG-3′<br>5′-AGAGCTCTTCCCTTCCTTCC-3′
Tet2	5′-CTTCTCTGCTCATTCCCACA-3′<br>5′-AGCTCCGACTTCTCGATTGT-3′
Tet3	5′-CAGCGATTGTCTTCCTTGGT-3′<br>5′- GCCTGCATGGACTTCTGTG-3′
Spcs3	5′-CGTCTCGCGGATCATGCTAAA-3′<br>5′-AGC TTCGGATTATCACCTCTCA-3′
Jph2	5′-AACGACATGGGCTTGGCATA-3′<br>5′-GATAGGTTCCTCCGTCTGCG-3′
Vapb	5′-GGGCCTCTCTCAATGTGTCT-3′<br>5′-CCTCCTTCCATACTGCCTCC-3′
Ipmk	5′-GATGGAGGAGATCGGGTTCC-3′<br>5′-GTGCTGGTTTTGTGTCTCGT-3′
Nppa	5′-GGGGGTAGGATTGACAGGAT-3′<br>5′-CAGAATCGACTGCCTTTTCC-3′
Nppb	5′-ACAAGATAGACCGGATCGGA-3′<br>5′-ACCCAGGCAGAGTCAGAAAC-3′
Gapdh	5′-GAGACGGCCGCATCTTCTT-3′<br>5′-CAATCTCCACTTTGC CACTGC-3′
